# Refractory Vasospastic Angina and Sudden Cardiac Arrest: Is Implantable Cardioverter Defibrillator Indicated and Is It Always Protective?

**DOI:** 10.7759/cureus.9613

**Published:** 2020-08-08

**Authors:** Sana Riaz, Vijay Raj, Siddharth Shah

**Affiliations:** 1 Internal Medicine, State University of New York Upstate Medical University, Syracuse, USA; 2 Cardiovascular Medicine, State University of New York Upstate Medical University, Syracuse, USA; 3 Cardiovascular Medicine, State University of New York Upstate Medical University, Syrcause, USA

**Keywords:** vasospastic angina, sudden cardiac arrest, implantable cardioverter defibrillator, ventricular tachycardia

## Abstract

Vasospastic angina (VSA) is characterized by episodic chest pain associated with transient ST-segment abnormalities on electrocardiogram, secondary to vasospasm of the epicardial coronary artery. We report the rare case of a 46-year-old female with refractory VSA secondary to multivessel coronary vasospasm causing an anterior myocardial infarction. She succumbed secondary to ventricular tachycardia (VT) storm, in spite of being on maximally tolerated medical therapy and having an implantable cardioverter defibrillator (ICD) for secondary prevention of VT. Contemporary guidelines recommend ICD implantation (class IIa) in VSA patients who survived sudden cardiac arrest (SCA), if they are already on optimal medical therapy or if medical therapy is not tolerated. Whether ICD implantation is appropriate in VSA patients with aborted SCA, even before assessing the response to medical therapy, is not well known and requires further studies.

## Introduction

Prinzmetal variant angina syndrome, first described in 1959, is characterized by episodic chest pain associated with transient ST-segment abnormalities, often ST elevation on the electrocardiogram (ECG). It is also known as ‘variant angina’ or ‘vasospastic angina (VSA)’ as transient vasospasm of the epicardial coronary artery plays an important role in its pathophysiology [1]. The prevalence is higher in Japan as compared to other parts of the world. We hereby present a rare case of a young female with refractory VSA causing an anterior myocardial infarction who succumbed secondary to ventricular tachycardia (VT) storm, in spite of being on maximally tolerated medical therapy and having an implantable cardioverter defibrillator (ICD) for secondary prevention of VT. 

## Case presentation

**Figure 1:**
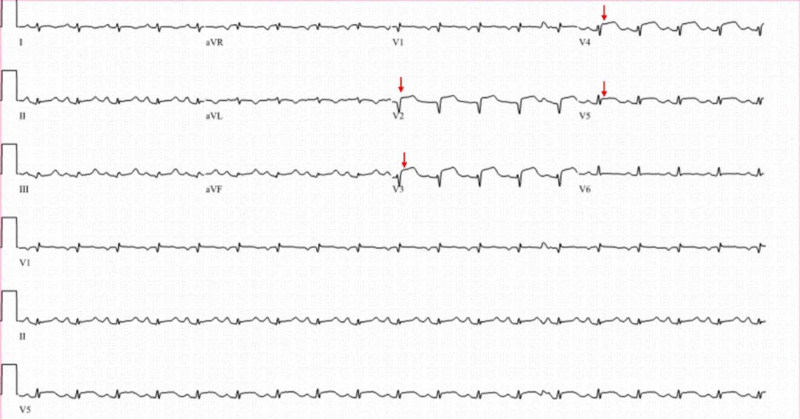
Electrocardiogram on admission showing ST-segment elevation (red arrows) in antero-lateral leads (V2-V5).

**Figure 2:**
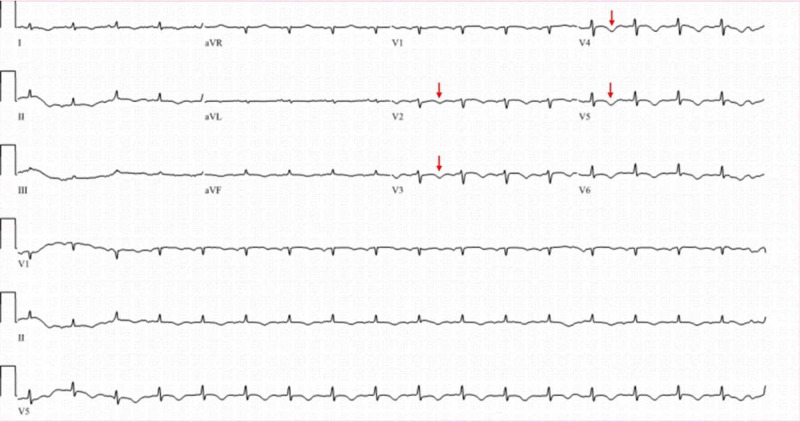
Repeat electrocardiogram showing resolution of ST-segment elevation and new T-wave inversion (red arrows) in leads V2 through V5.

A 46-year-old female presented following an out-of-hospital sudden cardiac arrest (SCA) secondary to ventricular arrhythmia. She had a past medical history of VSA, insignificant coronary artery disease (CAD), long-standing tobacco use, and hypertension. Her home medications were aspirin 81 mg daily, isosorbide mononitrate 60 mg daily, and diltiazem extended release 120 mg daily. She had return of spontaneous circulation (ROSC) following cardiopulmonary resuscitation (CPR) for 10 minutes, required defibrillation, and was subsequently intubated for acute encephalopathy. ECG showed ST-segment elevation in anterior leads (Figure 1), which resolved on repeat ECG (Figure 2). The initial troponin T was 0.29 ng/mL. She continued to have recurrent transient episodes of ST-segment elevation, as noted on telemetry. Echocardiogram showed a left ventricular ejection fraction (LVEF) of 25%-30% with hypokinesis of apical region. Urine toxicology screen was negative for any illicit drugs. 

**Figure 3:**
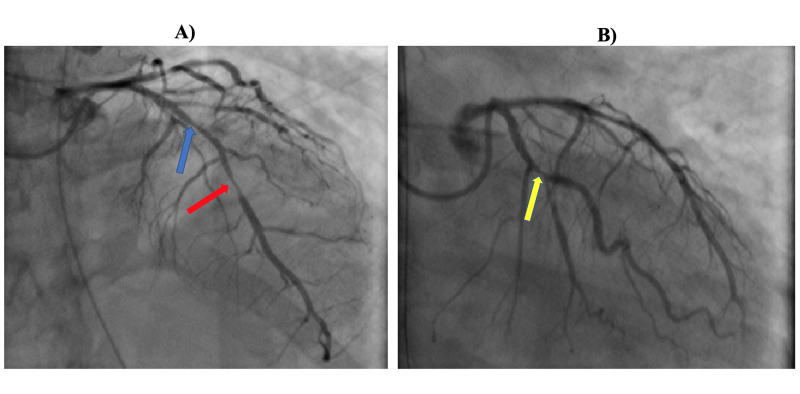
A) Coronary angiogram showing 60% stenosis of mid left anterior descending (LAD) coronary artery (blue arrow) and diffuse 90% stenosis of the distal LAD (red arrow). B) Coronary angiogram showing 70% stenosis of the obtuse marginal 1 (OM1) artery (yellow arrow).

**Figure 4:**
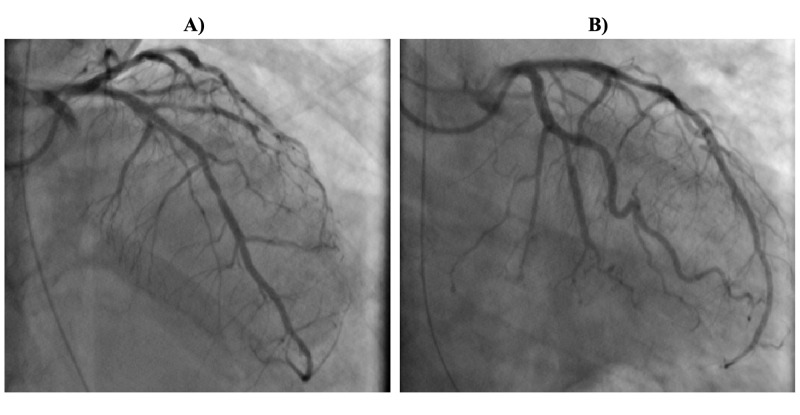
A) Coronary angiogram showing disappearance of lesion in the mid left anterior descending coronary artery and B) lesion in the first obtuse marginal coronary artery, following administration of intracoronary nitroglycerin, thus confirming coronary vasospasm.

She underwent emergent coronary angiogram, which showed 60% lesion of mid left anterior descending (LAD) coronary artery, 90% diffuse stenosis of the distal LAD, and 80% lesion of the obtuse marginal 1 (OM1), while rest of the coronary arteries were angiographically normal (Figure 3). Intracoronary nitroglycerin was administered, and repeat angiogram showed normalization of LAD and OM1 stenosis, thus confirming the diagnosis of coronary vasospasm (Figure 4). Following the procedure, she continued to have recurrent episodes of transient ST-segment elevation, as seen on telemetry, while she remained on IV nitroglycerin. She subsequently had another episode of SCA. The initial rhythm was VT followed by pulseless electrical activity, and she required 30 minutes of CPR. She was eventually extubated and transitioned to oral isosorbide mononitrate 90 mg once daily and diltiazem extended release 360 mg daily. Repeat echocardiogram showed normalization of LVEF with resolution of wall motion abnormalities. She underwent an ICD implantation for secondary prevention of SCD. 

She was followed periodically in the outpatient clinic, and ICD interrogation at one and three months did not reveal any further ventricular arrythmias. Unfortunately, she suffered another episode of SCA at home secondary to VT storm, at six months following discharge and multiple ICD shocks failed to revive her. 

## Discussion

VSA can have a wide spectrum of clinical presentation from no symptoms to SCA from VT or ventricular fibrillation [2]. Common risk factors for VSA include smoking, hyperlipidemia, magnesium deficiency, physical or emotional stress, sympathomimetic agents, and hyperventilation. While diagnosis is often challenging, Holter monitoring (which can reveal transient ST-segment elevation with associated symptoms) and provocative testing in the cardiac catheterization laboratory are often useful in providing definitive diagnosis and identifying various forms of arrhythmias [1]. Management of VSA includes avoiding precipitating factors such as smoking and vasodilator therapy with either long-acting calcium channel blockers (CCBs) or long-acting nitrates, while a combination therapy may be needed in severely symptomatic cases as seen in our patient [3]. Additional agents that may have a potential benefit but have a limited evidence include nicorandil, statins, aspirin, magnesium, vitamins C and E, Iloprost, alpha receptor blockers, selective serotonin receptor inhibitors, and selective thromboxane A2 synthetase inhibitors. 

While VSA has a good long-term prognosis, data regarding the prognosis and management of patients with aborted SCA (ASCA) remain controversial. Takagi et al. showed that among 35 VSA patients with ASCA, from a group of 1,429 patients with VSA, there were two ICD shock treatments and one SCA over a mean follow-up of 2.7 years [4] . However, another study did not show any cardiac events among 17 patients with ASCA, over a mean follow-up of 5.6 years [5]. A recent larger study by Ahn et al. including 188 VSA patients with ASCA and 1844 VSA patients without ASCA showed significantly higher rates of cardiovascular mortality and all-cause mortality in VSA patients with ASCA, when compared to VSA patients without ASCA [6] . It was also noted that VSA patients with ASCA had a high incidence of recurrent ventricular arrhythmias. Similarly, another study also demonstrated high risk of recurrent ventricular arrhythmias in patients with ASCA secondary to VSA despite medical therapy. Thus, ICD may be protective, especially in this high-risk group of patients with ASCA [7]. 

Contemporary guidelines recommend ICD implantation with a class IIa recommendation in VSA patients who survived SCA, while on optimal vasodilator therapy or in whom medical therapy is not tolerated [8]. Our patient suffered SCA despite being on two vasodilators and thus received ICD implantation for secondary prevention of SCA. VSA patients who survive SCA continue to have high recurrence of ventricular arrhythmias or SCA in spite of intensive vasodilator therapy [6]. Whether ICD implantation is appropriate in VSA patients with ASCA, even before assessing the response to medical therapy, is not well known, and thus, it currently remains a class IIb recommendation [8]. While ICD is generally effective in terminating ventricular arrhythmias secondary to coronary vasospasm, refractory ventricular arrhythmias not responsive to ICD therapy can very rarely happen, as unfortunately seen in our case and is likely a result of multivessel coronary spasm. 

## Conclusions

Our case highlights a rare presentation of refractory VSA secondary to multivessel coronary vasospasm with resultant anterior myocardial infarction, which did not respond to conventional vasodilators and raises few important points. First, additional medical therapy, other than nitrates and CCBs, may be beneficial as mentioned above, especially in refractory cases like ours, and requires further studies to evaluate the same. Second, ICD implantation may be beneficial in VSA patients with ASCA even before the trial of medical therapy as the risk of recurrent ventricular arrhythmias is high, as also noted in our patient. Third, ICD therapy may not always be effective in preventing SCA secondary to ventricular arrhythmias. 
